# Impostor syndrome and burnout among American medical students: a pilot study

**DOI:** 10.5116/ijme.5801.eac4

**Published:** 2016-10-31

**Authors:** Jennifer A. Villwock, Lindsay B. Sobin, Lindsey A. Koester, Tucker M. Harris

**Affiliations:** 1 Department of Otolaryngology, University of Kansas Medical Center, Kansas City, USA; 2 Department of Otolaryngology, Boston Children's Hospital, Boston, MA, USA; 3 Department of Otolaryngology, University of Pittsburgh Medical Center, Pittsburgh, PA, USA; 4 Arnot Medical Services, Arnot Ogden Medical Center, Elmira, NY, USA

**Keywords:** Impostor syndrome, American medical students, pilot study, USA

## Abstract

**Objectives:**

To describe levels of burnout and impostor syndrome (IS) in medical students, and to recognize demographic differences in those experiencing burnout and IS.

**Methods:**

Research participants included 2,612 medical students who entered Jefferson Medical College between 2002 and 2012. This sample was divided into two groups: Matriculants between 2002 and 2007 (n=1,380) and between 2008 and 2012 (n=1,232). Data for 2002-2007 matriculants were subjected to EFA (principal component factor extraction), and data for matriculants of 2008-2012 were used for CFA (structural equation modeling, and root mean square error for approximation).

**Results:**

One hundred and thirty-eight students completed the questionnaire. Female gender was significantly associated with IS (χ^2^_(3)_=10.6, p=0.004) with more than double the percentage of females displaying IS than their male counterparts (49.4% of females versus 23.7% of males). IS was significantly associated with the burnout components of exhaustion (χ^2^ _(2)_=5.9, p=0.045), cynicism (χ^2^_(2)_=9.4, p=0.004), emotional exhaustion (χ^2^_(2)_=8.0, p=0.018), and depersonalization (χ^2^ _(2)_=10.3, p=0.006). The fourth year of medical school was significantly associated with IS (χ^2^_(3)_=10.5, p=0.015).

**Conclusions:**

Almost a quarter of male medical students and nearly half of female students experience IS and IS was found to be significantly associated with burnout indices. Given the high psychological morbidity of these conditions, this association cannot be ignored. It behooves us to reconsider facets of medical education (i.e. shame-based learning and overall teaching style) and optimize the medical learning environment.

## Introduction

Physician burnout has received a considerable amount of attention in recent years. Medicine self-selects for individuals who are driven, competitive, and able to endure years of intense schooling and high expectations. Unfortunately, these qualities also contribute to the development of burnout, the ramifications of which go beyond the individual experiencing it. Among residents, burnout is associated with absenteeism and, more disturbingly, medical errors and self-reported delivery of suboptimal care.^1^

A related, but less investigated entity which has gained recent popularity in the lay press and scientific literature is impostor syndrome (IS) or impostorism.^2^^-^^5^ IS is characterized by chronic feelings of self-doubt and fear of being discovered as an intellectual fraud. Despite evidence of abilities, those suffering from IS are unable to internalize a sense of accomplishment, competence, or skill. Overall, they believe themselves to be less intelligent and competent than others perceive them to be.^6^ A 1998 study by Henning et al found that among medical, dental, nursing, and pharmacy students, 30% scored as impostors. Within this population, IS was found to be the strongest predictor of general psychological distress. A similar study by Oriel et al found imposterism present in approximately one third of their sample of family medicine residents. This was despite the fact that all the respondents felt they were receiving the training necessary to succeed in their careers.^7^

IS has several potential implications for medical education. Those suffering from IS are less likely to speak up or volunteer answers and information than their unaffected peers. This may lead to innate differences in learning style and a subsequent need to tailor curriculum to take into account the large proportion of learners with IS. Furthermore, the literature largely supports a higher levels of IS in women.^6^ With women composing approximately 47% of medical school classes, IS may have far reaching impacts, such as overall psychological health in students, specialty selection, and a potential to shift current educational paradigms.  In fact, prior studies have shown that program year and progression in training do not have an impact on IS, indicating that IS may be a static trait and not able to be “trained out” of students.^6^

IS, especially in relation to burnout, remains an area in need of further investigation among the medical student population. This pilot study seeks to explore IS and burnout in medical students and the associated comorbidity of burnout. 

## Methods

### Participants

This pilot study’s protocol was reviewed and approved with exempt status by the State University of New York – Upstate Medical University Institutional Review Board. This study was received and determined to be of exempt status by the SUNY-Upstate Medical University Institutional Review Board because of its educational nature and the fact that it exposed participants to no risk. From November 2013 to March 2014, medical students from a single United States medical school received an e-mail invitation to participate in an anonymous, self-administered, web-based survey. One hundred and thirty-eight medical students completed this pilot survey (response rate of 20%). Responses were received from students across all training years. Demographic data is shown in Table 1.

**Table 1:** Demographic data of participants (N=138)

Variable		Respondents N (%)
Age		
	18 – 24	59 (42.75)
	25 – 30	69 (50)
	31 – 35	5 (3.62)
	36 – 40	2 (1.45)
	41 – 45	1 (0.72)
	46 – 50	2 (1.45)
Female		77 (56.2)
Race		
	Black	2 (1.46)
	White	116 (84.67)
	Latina/o	4 (2.92)
	Asian	16 (11.68)
	Native American	1 (0.73)
	Multiracial	4 (2.92)
Year of Training		
	1^st^ year	41 (29.71)
	2^nd^ year	29 (21.01)
	3^rd^ year	34 (24.64)
	4^th^ year	34 (26.64)
Plans to pursue fellowship training		
	Yes	55 (39.86)
	No	16 (11.59)
	Undecided	67 (48.55)
Worked outside of medicine > 1 year		
	Yes	67 (48.91)

Students in all levels of training responded at roughly similar rates. There was a slight female preponderance of responses (56.2%). The vast majority of students identified as Caucasian (84.6%) with Asian being the next most common race (11.68%). Nearly half of medical students in our cohort have spent at least one year working in a field outside of medicine. Future specialty choice was highly variable, with many students undecided.

### Data collection

Demographic data collected from participants included age range, race, gender, year in training, desired specialty, and if there had been a gap in educational training for work in a field outside of medicine for greater than one year. 

The Young Impostor Scale (YIS) was used to dichotomously assess for the presence or absence of IS. The YIS is an eight item instrument used to assess for impostor-like feelings (e.g. do you secretly worry that others will find out that you’re not as bright and capable as they think you are?” See Appendix 1). Responding “Yes” to five or more of these questions was considered a positive finding of IS.

As described by Maslach et al, the Maslach Burnout Inventory - Human Services Survey was used. This questionnaire evaluates the frequency with which respondents experience feelings across the domains of emotional exhaustion, depersonalization, and personal accomplishment.^8^^-^^9^ It is the most commonly used instrument for measuring burnout and has been demonstrated to have good psychometric properties and its validity confirmed in multiple studies.^8^^,^^10^ Respondents rate statements on a scale of zero (never) to six (every day). Sample statements include “I feel emotionally drained from my work,” “I don’t really care what happens to some patients,” and “I feel I’m positively influencing other people’s lives through my work.” Using normative values for medical professionals, burnout was determined to be high if the following scores were met or exceeded: Emotional exhaustion (> 27 points), depersonalization (> 10 points), and personal accomplishment (< 33 points).^9^

### Statistical analysis

All statistical calculations were performed using SPSS Version 17 software (IBM Corporation, Armonk, NY, USA). Data was automatically collected by the internet-based survey software. The data were then imported into SPSS for analysis. The presence of IS and burnout components were analyzed across age, gender, race, year of training, intention to pursue fellowship training, and greater than one year of work experience outside of medicine using chi-squared tests. The association between burnout and IS was also compared using chi-squared tests. Significance was set at p < 0.05.

## Results

Female gender was not significantly associated with burnout components. However, female gender was significantly associated with IS (χ^2^_(3)_ = 10.6, p = .004), with more than double the percentage of females displaying IS than their male counterparts (49.4% of females versus 23.7% of males).

There was a trend towards significance between race and IS. The groups that make up the majority of medical students – Asians and whites – have a 30% rate of IS, versus 72.7% in all other races (χ^2^_(3)_ = 6.87, p = .057). The majority of students were undecided with respect to their specialty, precluding analysis of specialty choice on burnout or IS.

IS was significantly associated with multiple burnout components. These included exhaustion (χ^2^_(2)_ =5.9, p=0.045), cynicism (χ^2^_ (2)_ =9.4, p=0.004), emotional exhaustion (χ^2^_(2)_=8.0, p=0.018), and depersonalization (χ^2^_ (2) _=10.3, p=0.006), see Table 2 Only the fourth year of medical school was significantly associated with IS (χ^2^_(3)_ =10.5, p = 0.015). Age > 24 years old was significantly associated with lower levels of personal accomplishment (χ^2^_ (2)_ =6.87, p = 0.031).

**Table 2:** Each burnout component is stratified by presence or absence of IS (N=138)

Burnout Components		Degree of Burnout N (%)		
		High	Moderate	Low
Depersonalization^*^				
	IS	14 (27)	18 (35)	20 (38.5)
	No-IS	7 (8.1)	28 (33)	51 (59)
Personal Accomplishment				
	IS	8 (15)	19 (37)	25 (48)
	No-IS	16 (19)	25 (48)	50 (58)
Emotional Exhaustion^*^				
	IS	25 (48)	19 (37)	8 (15)
	No-IS	23 (26)	35 (40)	28 (32)
Professional Efficacy				
	IS	1 (2)	4 (8)	47 (90)
	No-IS	0 (0)	5 (6)	81 (94)
Exhaustion^*^				
	IS	40 (77)	12 (23)	0 (0)
	No-IS	50 (58)	33 (38)	3 (3.5)
Cynicism^*^				
	IS	36 (69)	15 (29)	1 (1.9)
	No-IS	15 (29)	46 (54)	0 (0)

*The burnout component is significantly associated with IS (p<0.05).

## Discussion

This pilot study sought to explore IS and burnout in medical students. In our survey of 138 medical students, we found that female gender was significantly associated with IS. IS was significantly associated with burnout; students with IS had significantly increased levels of exhaustion, emotional exhaustion, cynicism, and depersonalization. Clearly, these are not the traits medical education seeks to foster.

The results of our pilot study are supported by prior published studies. Oriel et al similarly found that gender impacted IS with 41% of female, and only 23% of male, family medicine residents experiencing IS.^7^ The gender discrepancy in IS levels has implications for medical education as coping mechanisms also differ. For example, women tended to manage IS by facing their doubts and competing harder; men tended to avoid areas in which they felt vulnerable.^11^ A survey of medical, dental, nursing, and pharmacy students by Henning et al also found a similar level – 30% - of IS in their respondents. Interestingly, in a cohort of 255 family medicine residents wherein one-third experienced IS, almost all indicated their training was adequate preparation for a successful career. However, those with IS suffered more psychological distress and still worried that they would not be ready for practice after graduation.^7^ While we did not assess for depression and anxiety in our cohort, prior studies have shown a link between IS and depression, anxiety, and self-esteem issues.^6^^,^^7^

Prior studies have not shown an association between program year and IS - indicating that impostorism may be a static behavioral trait.^6^^-^^7^ In contrast, we found that fourth year medical students experienced a significant increase in IS. This may be due to the students preparing for and participating in the Matching process for residency, a highly competitive and stressful time. This is consistent with the suggestion by Clance that impostor feelings are most pronounced when faced with new challenges.^6^^,^^12^ As the student progresses through training, they assume more responsibility and autonomy. For the medical student, this culminates in the first day of intern year, when they are called “doctor” but typically do not feel like the knowledgeable physician they desire to be.^7^ In addition to the stresses associated with the matching process, it is possible that fourth year medical students feel IS more acutely than in prior years because they are closest to their first day as a doctor.

In contrast to IS, burnout in medicine has received much greater attention. Burnout is most commonly defined as a triad of emotional exhaustion, depersonalization, and diminished feelings of personal accomplishment. The typical picture of burnout mimics depression and is all too familiar in medicine: fatigue, inability to concentrate, insomnia, irritability, and feeling as if one is “just going through the motions”.^1^ Data from prior studies has shown that 50% of those who met the criteria for burnout also met criteria for depression.^13^ Among practicing physicians, 46-80% reported moderate to high levels of emotional exhaustion, 22-93% reported moderate to high levels of depersonalization, and 16-79% reported low to moderate levels of personal achievement. Disturbingly, these results are similar to those previously reported for medical students.^14^ These figures are in agreement with our results, which revealed high levels of most burnout indices among medical students.

The psychological comorbidities of IS and burnout cannot be ignored. Henning et al discovered that 30% of medical, dental, nursing, and pharmacy students met IS criteria and suffered increased psychological distress.^15^ In a study of 4287 medical students, Dyrbye et al reported that 49.6% had experienced burnout and that 11.2% had struggled with suicidal ideation in the past year.^16^ Burnout and low mental quality of life were independent risk factors of suicidal ideation.^17^ These risk factors for suicide translate into medical practice where male physicians are twice as likely, and female physicians three times as likely, to commit suicide than the general public.^18^ Perhaps most disturbing is that multiple studies have shown that while medical students begin their training with similar rates of depression as their nonmedical counterparts,^17^ their mental health declines as their education progresses.^17^^,^^19^^,^^20^ This indicates that the beginning stages of burnout first emerge in the earliest stages of physician training.

Limitations of this study include that it is a self-administered, survey based, convenience sample of medical students at one United States medical school with a relatively small sample size. Significant associations may change with a larger sample. The self-reporting and self-selection of participants introduced bias with many survey items subject to social desirability response bias. We did try to minimize this bias with the anonymous nature of the survey. Additionally, our results are similar to those presented by other authors and it has been previously shown that the MBI is resistant to the threat of social desirability bias.^9^ Another limitation is the cross-sectional design of the study. This does not allow the establishment of causal relationships between variables. Also, the experience of burnout and contributing factors are likely to be highly individualized. This study did not assess potential contributing factors to IS and burnout. Additional limitations include the lack of depression and anxiety assessment. These measurements would have added significant time to survey completion, and were excluded in order to increase student participation. An additional limitation is the lack of normative data for the Young Impostor Scale. A normal or acceptable level of IS has not been delineated.  

Assuming the medical students surveyed in our pilot study are representative of medical students in the United States, we submit that optimization of the training paradigm to take into account IS and burnout is needed.  Ensuring the wellbeing of our future physicians must be a priority. The core of medical education essentially remains unchanged since the Flexner report in the early 1900s. Specific suggestions have been made such as regular, timely, and positive feedback from instructors; regular feedback is now a metric required by the ACGME. However, with the large numbers of trainees struggling with IS, burnout, and the associated psychological comorbidities, a shift away from the traditional “shame-based” learning and “pimping” to more of an open and consistent educational dialogue may be needed. Additionally, given the high numbers of medical professionals experiencing psychological stressors such as burnout and IS, it is important that attention also be paid to discovering ways to counter these negative influences and enhance wellness. For example, professional development programs, beginning early in medical training, may be of benefit to help foster development of overall self-efficacy.^21^ Additionally, strong mentoring relationships that allow expression of professional and personal concerns without fear of repercussions have been shown to lower levels of burnout in surgical residency.^22^ 

Future research will focus on identifying medical students at risk for burnout, IS, and associated psychosocial comorbidities, while identifying ways to ameliorate the negative impact of these conditions.

## Conclusions

IS exists in a significant percentage of medical students and appears to peak in the fourth year of medical school.  Additionally, it is associated with multiple burnout indices and, likely, psychological distress. Further discussion regarding medical education paradigms in light of high levels of burnout and IS is needed.

### Conflict of Interest

The authors declare that they have no conflict of interest.

## Supplementary material

Supplementary file 1 Appendix 1. Young Imposter Syndrome Quiz (“Yes” or “No”)
