# Supplementary material for: Impostor syndrome and burnout among American medical students: a pilot study
Source: Int J Med Educ. 2016 Oct 31;7:364–9. doi: 10.5116/ijme.5801.eac4 (PMC5116369; doi:10.5116/ijme.5801.eac4)
Supplement: Supplementary file 1 — Appendix 1. Young Imposter Syndrome Quiz (“Yes” or “No”) [file ijme-7-364-S1.pdf]

Appendix 1.

Young Imposter Syndrome Quiz ("Yes" or "No")

1. Do you secretly worry that others will find out that you're not as bright and capable as they think you are?
2. Do you sometimes shy away from challenges because of a nagging self-doubt?
3. Do you tend to chalk your accomplishments up to being a "fluke," "no big deal" or the fact that people just "like" you?
4. Do you hate making a mistake, being less than fully prepared, or not doing things perfectly?
5. Do you tend to feel crushed even by constructive criticism, seeing it as evidence of your "ineptness?"
6. When you do succeed, do you think "Phew, I fooled them this time, but I may not be so lucky next time?"
7. Do you believe that other people (students, colleagues, competitors) are smarter and more capable than you?
8. Do you live in fear of being found out, discovered, or unmasked?
